# Quasi-bound states in the continuum with a stable resonance wavelength in dimer dielectric metasurfaces

**DOI:** 10.1515/nanoph-2023-0166

**Published:** 2023-05-01

**Authors:** Shaojun You, Mimi Zhou, Lei Xu, Deliang Chen, Menghui Fan, Jing Huang, Wenbin Ma, Shengyun Luo, Mohsen Rahmani, Chaobiao Zhou, Andrey E. Miroshnichenko, Lujun Huang

**Affiliations:** School of Physics and Mechatronic Engineering, Guizhou Minzu University, Guiyang 550025, China; School of Chemical Engineering, Guizhou Minzu University, Guiyang 550025, China; Advanced Optics and Photonics Laboratory, Department of Engineering, School of Science Technology, Nottingham Trent University, Nottingham NG11 8NS, UK; School of Physics and Electronic Science, Guizhou Education University, Guiyang 550025, China; School of Materials Science and Engineering, Guizhou Minzu University, Guiyang 550025, China; School of Engineering and Information Technology, University of New South Wales at Canberra, Northcott Drive, Canberra, ACT 2610, Australia; The Extreme Optoelectromechanics Laboratory (XXL), School of Physics and Electronic Sciences, East China Normal University, Shanghai 200241, China

**Keywords:** all-dielectric metasurfaces, bound states in the continuum, stable resonance wavelengths

## Abstract

Symmetry-protected bound states in the continuum (SP-BICs) are one of the most intensively studied BICs. Typically, SP-BICs must be converted into quasi-BICs (QBICs) by breaking the unit cell’s symmetry so that they can be accessed by the external excitation. The symmetry-broken usually results in a varied resonance wavelength of QBICs which are also highly sensitive to the asymmetry parameters. In this work, we demonstrate that QBICs with a stable resonance wavelength can be realized by breaking translational symmetry in an all-dielectric metasurface. The unit cell of metasurface is made of a silicon nanodisk dimer. The Q-factor of QBICs is precisely tuned by changing the interspacing of two nanodisks while their resonance wavelength is quite stable against the interspacing. We also find that such BICs show weak dependence on the shape of the nanodisk. Multiple decompositions indicate that the toroidal dipole dominates this type of QBIC. The resonance wavelengths of QBICs can be tuned only by changing either the lattice constants or the radius of nanodisk. Finally, we present experimental demonstrations on such a QBIC with a stable resonance wavelength. The highest measured Q-factor of QBICs is >3000. Our results may find promising applications in enhancing light–matter interaction.

## 1 Introduction

All-dielectric metasurfaces have received tremendous attention in the past decades due to their extraordinary optical properties [1, 2]. Similar to metallic nanostructures supporting plasmonic resonances, they provide multipolar electric and magnetic resonances known as Mie resonances. On the other hand, they have lower intrinsic losses than their counterparts of noble metals. These two unique properties render them become ideal platforms for enhancing light–matter interactions and developing high-performance photonic devices. For example, they have been used to realize metalens with high efficiency [3–5], lasing [6–8], sensing [9–11], and enhanced generations [12–15]. At the early stage, much attention has been paid to the low-order Mie resonances with relatively low-quality factors (Q-factors), such as electric and magnetic dipole resonances. The moderate Q-factor of Mie resonance is always accompanied by moderate electric field confinement, which may limit light–matter coupling strength.

Recently, all-dielectric metasurfaces have been demonstrated to support optical bound states in the continuum (BICs) with infinite Q-factors [16–24]. BICs are the eigenstates with forbidden radiation, which are perfectly decoupled from the exterior environment though they are localized within the continuum [25–42]. Typically, they must be first converted into quasi-BICs (QBICs) with finite but still high Q-factors so that they can be excited by external sources, such as plane wave incidence. Among a diverse family of BICs, symmetry-protected BICs are the most intensively studied because they can be easily found in the dielectric metasurface whose unit cell follows certain symmetry. Usually, QBICs are induced by introducing in-plane symmetry broken, such as removing or adding parts of nanodisk [9, 14, 16, 27, 43], [44], [45], [46], [47], [48], [49], [50], [51]. The Q-factors of QBICs are deliberately tailored by the asymmetry parameters. Nevertheless, their resonance wavelengths are also tuned with the increasing asymmetry parameters. In some applications, creating a QBIC with a tunable Q-factor but a stable resonance wavelength is desirable.

In this work, we demonstrate that a dimer-based dielectric metasurface supports toroidal dipole (TD) dominated BICs. QBICs are realized by changing the interspacing between nanodisk dimers instead of removing or adding parts of the nanodisk to create the reflection symmetry-broken. Compared with previous symmetry-broken induced QBICs, such BICs have almost constant resonance wavelength, while their Q-factors are tuned carefully by controlling the interspacing between dimers without resorting to breaking symmetry. Also, we found that such BICs are independent on the shape of nanodisk. This may relax the fabrication tolerance. The resonance wavelengths of BICs or QBICs are engineered by either changing the lattice constants of the metasurface or changing the radius of the nanodisk for the dimer system. Finally, we experimentally demonstrated such QBICs by fabricating a series of silicon-dimer metasurfaces and measuring their transmission spectra. The vanished linewidth of Fano resonance verifies the emergence of BICs, and QBICs are characterized by high-Q Fano resonances. The retrieved Q-factors are as high as 3142. Our results may find promising applications in designing high-performance photonic devices based on QBICs with high-Q factors, such as lasers, biosensors, filters, etc.

## 2 Results and discussion

**Figure 1:**
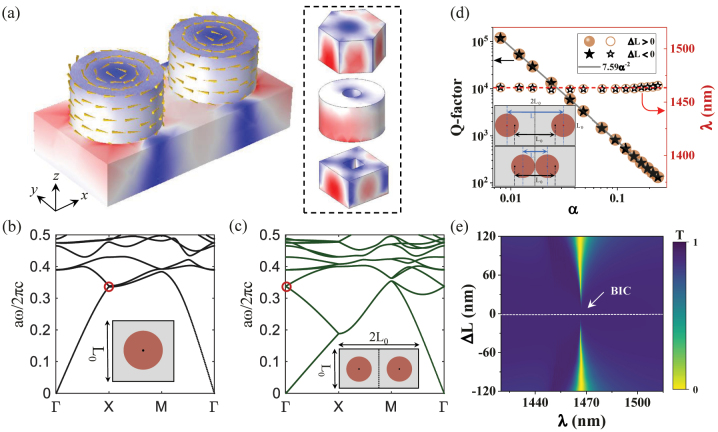
The excitation of quasi translational-symmetry-protected BICs. (a) The schematic of the unit cell of the proposed metasurface. The TD quasi-BIC can be excited by moving the relative position of dimer nanodisks, which is shape-independent. The circular nanodisks can be replaced by the hexagonal disks, circular or square hollow disks. Here, the color corresponds to the electric field intensity, the arrows describe the directions of displacement current. (b) and (c) The calculated bandgap structure for nanodisk arrays whose unit cell is 1 × 1 and 2 × 1. Here, *a* = *L*_0_. (d) Log–log plot of radiative Q-factor and resonance wavelength as a function of the perturbation parameter *α*. The inset shows a schematic diagram of the position perturbation of the dimer nanodisks with a period *L*_0_ = 500 nm in the *y*-direction and 2*L*_0_ = 1000 nm in the *x*-direction. *L* represents the distance between the centers of the two nanodisks. (e) The transmission mapping versus both the offset distance (Δ*L*) and resonance wavelength (*λ*).

To obtain a QBIC with a stable resonance wavelength, we first perform a design of moving the relative position of the meta-atom to disturb the translational-symmetry-protected BIC mode. The silicon dimer nanodisks are placed on the SiO_2_ substrate with a rectangular lattice (*L*_*x*_ = 2*L*_*y*_ = 2*L*_0_). The interspacing between two nanodisks is noted as *L*. BIC happens only at *L* = *L*_0_, the QBIC is induced when *L* deviates from *L*_0_ due to the broken translational symmetry of the unit cell, which is *L* > *L*_0_ or *L* < *L*_0_, as shown in Figure 1(a). Two circular displacement current distributions in opposite directions confirm that the toroidal dipole (TD) dominates such a QBIC. Compared to the single nanodisk array, the dimer, without disturbance, makes a band folding because of the reduced first Brillouin zone, inducing the emergence of BIC at Γ-point due to flipping guided modes at the band edge below the light cone [52]. Figure 1(b) and (c) show the transverse magnetic (TM) band structure of the metasurface whose unit cell is 1 × 1 and 2 × 1, it is clear that many new modes appear at Γ-point due to band folding from band edge *X* to Γ point. Herein, we set *L*_*x*_ = *L*_*y*_ = *L*_0_ = 500 nm for 1 × 1 unit cell and *L*_*x*_ = 2*L*_*y*_ = 2*L*_0_ = 1000 nm for 2 × 1 super unit cell. The refractive index of Si and SiO_2_ are *n*_Si_ = 3.48 and *n*_SiO2_ = 1.46, respectively. This work mainly focuses on the mode of normalized eigenfrequency *ωa*/2π*c* = 0.341, as shown by the red circle in Figure 1(b) and (c). The bandgap calculation is based on the open sources of Massachusetts Institute of Technology Photonic-Bands (MPB) [53]. We employ the commercial software COMSOL Multiphysics based on the finite element method to calculate the Q-factors of eigenmode. As illustrated in Figure 1(d), when the center distance *L* of the nanodisks deviates from *L*_0_, the BIC is converted to QBIC, and its *Q* ≈ 7.59*α* ^−2^, here the disturbance parameter *α* = $\frac{\left|\Delta L\right|}{L_{0}}=\frac{\left|L-L_{0}\right|}{L_{0}}$ . Therefore, the smaller the *α*, the larger the Q-factor. The schematic diagram of the disturbance of the device is shown in the inset of Figure 1(d). It is noted that the resonance wavelength of QBIC is almost fixed around 1464 nm with the change of *α*. To confirm the existence of such a BIC, we calculate the transmission spectrum mapping of the metasurface versus offset distance (Δ*L*) and incident wavelength under *y*-polarized light, as shown in Figure 1(e). Indeed, it can be seen that the resonance peak in transmission spectra shows the vanished linewidth at Δ*L* = 0 nm as BIC mode is perfectly decoupled to the plane wave incidence. As |Δ*L*| gradually increases, the resonance peak of the QBIC widens gradually, which corresponds to the decrease of Q-factor, as shown in Figure 1(d). These results demonstrate the existence of energy band folding BICs, which are also called artificial BICs.

**Figure 2:**
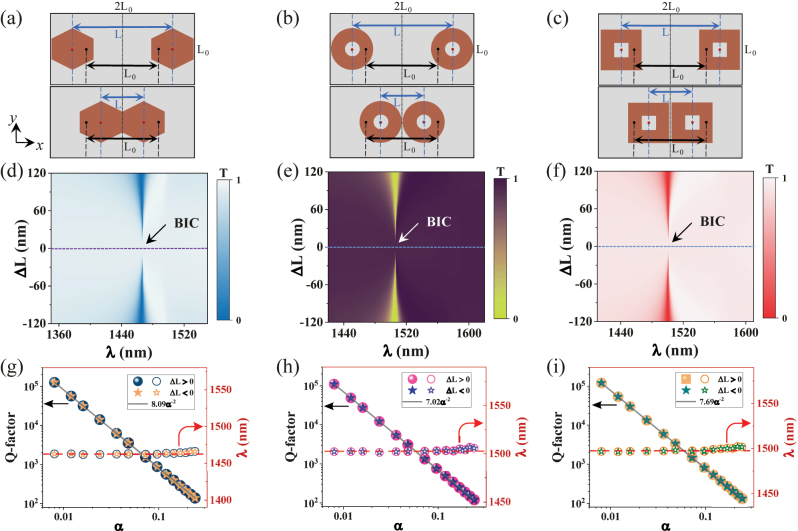
The optical responses of metasurfaces of hexagonal cube, circular or square hollow nanodisks. (a)–(c) Schematic diagrams of position perturbation, the hexagonal cube has a side length of 200 nm. The inner and outer radii of the circular hollow nanodisks are 55 nm and 190 nm. The inner and outer lengths of the square hollow nanodisks are 110 nm and 340 nm, respectively. The thickness of these metasurfaces is still 220 nm, and *L*_*x*_ = 2*L*_*y*_ = 2*L*_0_ = 1000 nm. (d)–(f) The transmission mappings versus both the offset distance (Δ*L*) and resonance wavelength. (g)–(i) The Q-factors and resonance wavelengths as a function of the perturbation parameter *α*.

Note that this BIC exhibits weak dependence on the shape of the nanodisk as long as the area of nanodisk in *x*o*y* plane is the same. For example, such a BIC mode always exists when the nanostructure is changed into a hexagonal cube, circular or square hollow nanodisks with nearly identical volume. Figure 2(a)–(c) show the schematic drawing of three types of nanodisk dimers with perturbation. The transmission spectrum mappings present the same BIC characteristics disappeared resonance peaks at Δ*L* = 0. When |Δ*L*| is larger than zero, the BICs are converted into QBICs with finite Q-factors, and the resonance spectra are gradually broadened as |Δ*L*| increases, suggesting reduced Q-factors. The calculated Q-factors of QBICs under different perturbation parameters *α* are exhibited in Figure 2(g)–(i), and the Q-factor are all proportional to *α* ^−2^ (*Q* = *Cα* ^−2^). Comparatively, the hexagonal cube metasurface has a larger coefficient of *C* = 8.09, which means that it holds a higher Q-factor for the same *α* value. Notably, the resonance wavelengths of QBICs of the three structures are also very stable. The wavelength of the hexagonal cube is located at 1463.5 nm, which is close to the result of the nanodisks studied above. The resonance wavelengths of circular or square hollow nanodisks are 1503 nm and 1498 nm, respectively, and they are close to each other. It is noted that the resonance wavelength of the QBIC without substrate is still stable. In this study, we almost fix the device’s volume, and the coupling difference between unit cells makes some difference in the resonance wavelengths of solid and hollow nanodisk metasurfaces. Therefore, such a BIC mode is robust against the device shape and exhibits large fabrication tolerances. In addition, the QBIC is excited by changing the relative position of the nanodisks, and their Q-factors only depend on the interspacing between nanodisks. In the fabrication process, the specific position parameters of the nanodisks are fixed, the samples are fabricated by electron-beam lithography (EBL) and inductively coupled plasma (ICP) etching techniques. The fabrication process only changes the uniformity and roughness of the nano-device, the position of the nanodisks does not move. Therefore, the perturbation parameter of QBICs is accurately controlled in the experiment, which provides a way to achieve an ultrahigh Q-factor in metasurfaces.

**Figure 3:**
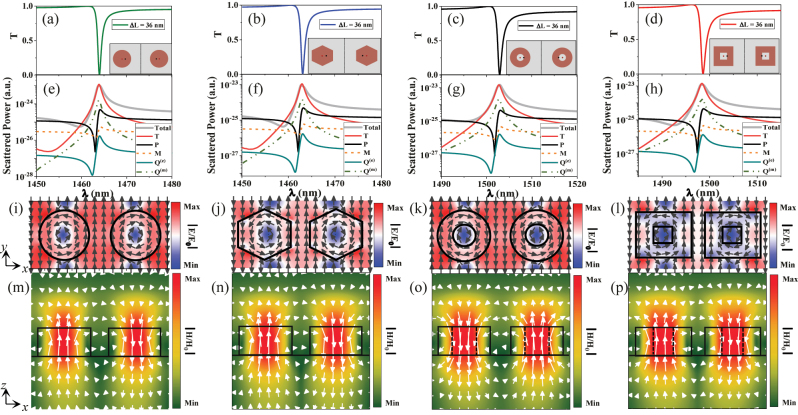
The demonstration of TD response of quasi-BIC. (a)–(d) The transmission spectra of the four structures at Δ*L* = 36 nm. (e)–(h) The total scattered power and contributions of dominant multipoles. (i)–(l) The electric field distributions in the *x*–*y* plane at the resonant wavelength. (m)–(p) Corresponding magnetic field distributions in the *x*–*z* plane. The black lines denote the boundaries of the nanostructure region. Black and white arrows refer to the electric field and magnetic field vector directions, respectively. The color scale corresponds to the field intensity.

Next, multiple decomposition is performed to reveal the nature of such a QBIC (see **Methods**). Here, we study the resonant properties of QBIC with Δ*L* = 36 nm as an example. Figure 3(a)–(d) show the transmission spectra of the four structures, and the asymmetric resonance peaks with the Fano profile are observed, which originate from the coupling between discrete bound states supported by dielectric nanodisks and continuum background. Figure 3(e)–(h) show the scattered power of multipole components. It is clear that the TD response dominates these modes. A subsequent contribution is a magnetic quadrupole (MQ), because pair of counter-oriented magnetic dipoles induces TD and MQ. The contributions of electric dipole (ED), magnetic dipole (MD) and electric quadrupole (EQ) are negligible. The electric and magnetic field distributions are exhibited in Figure 3(i)–(p). They share a similar field intensity distribution, thus verifying the same QBIC mode. It is clear that two reversed electric field vector distributions are observed in the region of the nanodisks at the *x*–*y* plane [see Figure 3(i)–(l)], which excite two magnetic dipoles with opposite directions, combining with the incident light field, and thus presenting a circular magnetic dipole distribution in the *x*–*z* plane [see Figure 3(m)–(p)], and finally excite a TD along the *y* direction.

We demonstrated above that this TD QBIC mode possesses a stable resonance wavelength. This can be understood because the resonance wavelength only depends on the effective dielectric volume when the coupling between super unit cells is fixed. Thus, there are two ways to adjust the resonance wavelength, one is to change the diameter of nanodisk, and the other is to change the lattice constants of metasurfaces.

**Figure 4:**
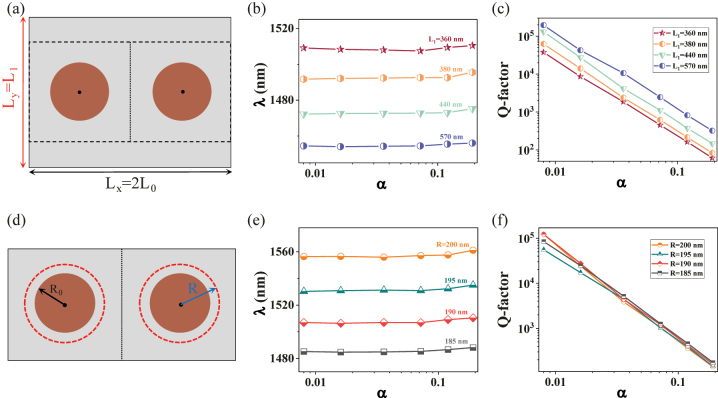
Two strategies for the tuning of stable resonance wavelength. (a) Schematic diagram of changing the size of the period in the *y*-direction, fixing *L*_*x*_ = 2*L*_0_, and changing *L*_*y*_ to *L*_1_. (d) Simultaneously changing the radius of the dimer nanodisks from *R*_0_ to *R*. (b), (e), (c), and (f) The resonance wavelengths and the Q-factors of QBICs versus perturbation parameters *α* for different *L*_1_ or *R* values.

Figure 4(a) shows the tuning way of changed lattice constants. Here *L*_*x*_ is fixed, and *L*_*y*_ is changed into *L*_1_, the calculated resonance wavelengths of QBICs are presented in Figure 4(b). As predicted, the wavelengths are still very stable, and they are adjusted from 1508 nm to 1454 nm when *L*_*y*_ is changed from 360 nm to 570 nm. The corresponding radiation Q-factors are shown in Figure 4(c). The same inverse quadratic dependence of Q-factors for QBICs excited by moving the position of nanodisks on the perturbation parameters *α* is observed. It is noted that the larger the *L*_*y*_, the larger the slope coefficient, which indicates that a larger *L*_*y*_ has a higher Q-factor for the same perturbation parameter. Another strategy is to simultaneously change the dimer nanodisk’s size simultaneously, as shown in Figure 4(d). One can find that a significant modulation of the resonance wavelength from 1485 nm to 1557 nm is observed when the nanodisk’s radius is changed from 185 nm to 200 nm. It is noted that the effect of the disk’s size on the Q-factor is smaller, especially for large values *α*. Therefore, we can design the structure parameters according to the target wavelength.

**Figure 5:**
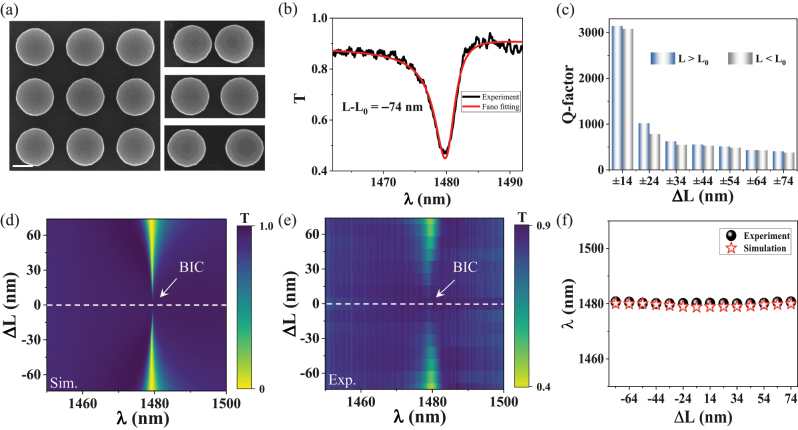
The experimental confirmation of QBICs with stable wavelength. (a) Top-view SEM images of the fabricated samples with different Δ*L*, the scale bar in white is 200 nm. (b) The measured transmission spectrum and fitted result from the Fano formula for Δ*L* = −74 nm. (c) The experimental Q-factors were calculated by $\frac{\omega_{0}}{\Gamma }$ . (d) and (e) The simulated and measured transmission spectrum mappings. The BIC phenomenon can be clearly observed at Δ*L* = 0. (f) Comparison between calculated and measured resonance wavelengths of QBIC.

Finally, we present the experimental demonstrations on such QBICs with a stable resonance wavelength. The samples are fabricated based on the 220 nm silicon-on-insulator (SOI) platform through EBL and ICP etching techniques (see **Methods**). Fifteen samples are fabricated with offset distance Δ*L* varying from −74 nm to 74 nm. The scanning electron microscopy (SEM) images of the fabricated sample for the cases Δ*L* = 0, Δ*L* < 0 and Δ*L* > 0 are shown in Figure 5(a). The measured transmission spectrum for Δ*L* = −74 nm is exhibited in Figure 5(b), we fit the resonance spectrum by the Fano formula [54, 55] $T\left(\omega \right)=T_{0}+A_{0}\frac{{\left[q+2\left(\omega -\omega_{0}\right)/\Gamma \right]}^{2}}{1+{\left[2\left(\omega -\omega_{0}\right)/\Gamma \right]}^{2}}$ . Where *ω*_0_ is the resonance frequency, Γdenotes the linewidth, *T*_0_ refers to the transmission offset, *A*_0_ means the continuum-discrete coupling constant, and *q* is the Breit–Wigner–Fano parameter determining asymmetry of the resonance profile. The Q-factor can be calculated by $\frac{\omega_{0}}{\Gamma }$ , and the experimental Q-factors are listed in Figure 5(c). As expected, the Q-factor increases as |Δ*L*| decreases, and the maximum experimental Q-factor reaches 3142. We compare the simulated and experimental transmission spectrum mappings, as shown in Figure 5(d) and (e), and the results are in good agreement. At |Δ*L*| = 0, the vanished resonance peak is indeed observed, further confirming the existence of bandgap folding BIC. We extract the measured and simulated resonance wavelengths, which are in very good agreement, as illustrated in Figure 5(f), and measured resonance wavelengths are indeed very stable. The experimental Q-factor is lower than the theoretical value, which may be contributed by the roughness, non-uniformity of the nanodisks and some incidence angles, which lead to strong scattering of light into free space. Higher Q-factors may be achieved by optimizing fabrication processes and characterization systems. In Table 1, we list the Q-factors of toroidal dipole resonance. Except for the stable TD resonance wavelengths, the Q-factor is also relatively high in this work. Thus, the BIC provides a new way for the realization of high Q-factor TD resonance.

**Table 1:** Comparison of Q-factors for toroidal dipole resonance.

Year	Nanostructures	Q-factors	Wavelength/frequency	References
2010	Metallic metamaterial	240	∼15.4 GHz	[56]
2016	Metallic metasurface	42.5	0.4 THz	[57]
2017	Metallic metasurface	18	∼200 GHz	[58]
2018	Metallic metasurface	29.8	0.15 THz	[59]
2018	Metallic metamaterial	12	4.16 GHz	[60]
2020	Dielectric metasurfaces	728	1505 nm	[61]
2020	Dielectric metasurfaces	160	∼700 nm	[61]
2019	Photonic crystal slabs	1373	1275 nm	[62]
2021	Dielectric metasurfaces	584	1500 nm	[63]
2021	Dielectric metasurfaces	206	756 nm	[64]
2022	Dielectric metasurfaces	261	1498 nm	[65]
2023	Dielectric metasurfaces	4990	1479 nm	[24]
2023	Dielectric metasurfaces	3142	1480 nm	In this work

## 3 Conclusions

In this work, we theoretically and experimentally demonstrate the QBICs with stable resonance wavelengths. An artificial BIC is observed in a silicon dimer nanodisk metasurface, arising from the band folding from the band edge below the light cone to point in the first Brillouin zone. The BIC is converted into a QBIC by breaking the translational symmetry of the unit cell. The resonance wavelength of QBIC is very stable because of the fixed volume of the metasurface and the stable coupling between the unit cells during the perturbation process. Meanwhile, the multipole decomposition and near-field distribution confirm that the TD response dominates such a QBIC. Moreover, this BIC is shape-independent no matter whether the nanodisk is a circular cylinder, a hexagonal cube, circular or square hollow disk. In addition, we confirm that the resonance wavelength of QBIC can be effectively regulated by changing the period and size of the nanodisks, so the on-demand resonance wavelength can be obtained by manipulating the structure parameters. Finally, we give experimental confirmation, and the maximum Q-factor can reach 3142 because of the weak shape dependence and the precise control of the disturbance parameters. Such a QBIC resonator with high Q-factors and stable resonance wavelengths is beneficial to the realization of high-quality nonlinear light source and low threshold laser.

## 4 Methods

### 4.1 Sample fabrication

The metasurfaces are fabricated on the silicon-on-insulator (SOI) wafer with 220 nm silicon of the top layer. Firstly, the ZEP-520A photoresist is spin-coated to the surface of a clear SOI wafer (1.5 cm × 1.5 cm). Subsequently, the metasurface patterns are defined into the photoresist by the electron-beam lithography (EBL) technique, and then developed and fixed. The silicon metasurface is etched by the inductively coupled plasma (ICP). Finally, the analytical solution removes the remaining photoresist in an ultrasonic bath.

### 4.2 Transmission measurements

The samples are measured by a piece of home-built equipment. The supercontinuum light source (YSL Photonics SC-5-FC, 400 nm–2200 nm) is applied, and the light is normally incident onto the fabricated metasurface, and then is collected by an optical spectrum analyzer (YOKOGAWA AQ-6370B, 600 nm–1700 nm).

### 4.3 Electromagnetic simulations

The Q-factors of eigenmode are calculated by the commercial software COMSOL Multiphysics based on the finite element method. The transmission spectra, field distributions, and multipole decomposition of metasurfaces are calculated by the finite-difference time-domain (FDTD) method. In the simulation, the periodical boundary conditions are employed in the *x-* and *y-*directions and perfectly matched layers (PML) are applied in the *z-*direction.

The multipole decomposition is employed to calculate the scattered power of multipole components. According to the current density ( $\vec{j}$ ) distribution in a unit cell, the multipole moments under the Cartesian coordinate can be obtained by the following formulas [62, 66]:

(1) $$electric\;dipole\;moment:\;\vec{P}=\frac{1}{i\omega }\int \vec{j}d^{3}r,$$

(2) $$magnetic\;dipole\;moment:\vec{M}=\frac{1}{2c}\int \left(\vec{r}\times \vec{j}\right)d^{3}r,$$

(3) $$toroidal\;dipole\;moment:\vec{T}=\frac{1}{10c}\int \left[\left(\vec{r}\cdot \vec{j}\right)\vec{r}-2r^{2}\vec{j}\right]d^{3}r,$$

(4) $$\begin{array}{ll} electric\;quadrupole\;moment:Q_{\alpha ,\beta }^{\left(e\right)} & =\frac{1}{2i\omega }\int \left[r_{\alpha }j_{\beta }+r_{\beta }j_{\alpha }\right.\\ & \;\left.-\frac{2}{3}\left(\vec{r}\cdot \vec{j}\right)\delta_{\alpha ,\beta }\right]d^{3}r, \end{array}$$

(5) $$\begin{array}{ll} magnetic\;quadrupole\;moment:Q_{\alpha ,\beta }^{\left(m\right)} & =\frac{1}{3c}\int \left[{\left(\vec{r}\times \vec{j}\right)}_{\alpha }r_{\beta }\right.\\ & \;\left.+\left({\left(\vec{r}\times \vec{j}\right)}_{\beta }r_{\alpha }\right)\right]d^{3}r, \end{array}$$

where *c* is the speed of light, *ω* is the frequency of light, and *α*, *β*, *γ* = *x*, *y*, *z*. And the far-field scattered power of these multipole moments can be calculated by the following formulas $I_{P}=\frac{2\omega^{4}}{3c^{3}}|\vec{P}{|}^{2}$ , $I_{M}=\frac{2\omega^{4}}{3c^{3}}|\vec{M}{|}^{2}$ , $I_{T}=\frac{2\omega^{6}}{3c^{5}}|\vec{T}{|}^{2}$ , $I_{Q^{\left(e\right)}}=\frac{\omega^{6}}{5c^{5}}\sum |Q_{\alpha ,\beta }^{\left(e\right)}{|}^{2}$ , $I_{Q^{\left(m\right)}}=\frac{\omega^{6}}{40c^{5}}\sum |Q_{\alpha ,\beta }^{\left(m\right)}{|}^{2}$ .

(6) $$\begin{array}{ll} & The\;total\;scattered\;power\;can\;be\;calculated\;from:\\ & \;I_{\text{total}}=I_{P}+I_{M}+\frac{4\omega^{5}}{3c^{4}}\left(\vec{P}\cdot \vec{T}\right)+I_{T}+I_{Q^{\left(e\right)}}+I_{Q^{\left(m\right)}}+o\left(\frac{1}{c^{5}}\right), \end{array}$$

where $\frac{4\omega^{5}}{3c^{4}}\left(\vec{P}\cdot \vec{T}\right)$ is the interference term of the electric and toroidal dipoles, and $O\left(\frac{1}{c^{5}}\right)$ has a small value.

## References

[1] Kuznetsov A. I., Miroshnichenko A. E., Brongersma M. L., Kivshar Y. S., Luk’yanchuk B. (2016). Optically resonant dielectric nanostructures. *Science*.

[2] Jahani S., Jacob Z. (2016). All-dielectric metamaterials. Nat. Nanotechnol..

[3] Khorasaninejad M., Chen W. T., Devlin R. C., Oh J., Zhu A. Y., Capasso F. (2016). Metalenses at visible wavelengths: diffraction-limited focusing and subwavelength resolution imaging. Science.

[4] Ou K., Yu F., Li G. (2021). Broadband achromatic metalens in mid-wavelength infrared. Laser Photonics Rev..

[5] Arbabi E., Arbabi A., Kamali S. M., Horie Y., Faraji-Dana M., Faraon A. (2018). MEMS-tunable dielectric metasurface lens. Nat. Commun..

[6] Ha S. T., Fu Y. H., Emani N. K. (2018). Directional lasing in resonant semiconductor nanoantenna arrays. Nat. Nanotechnol..

[7] Kodigala A., Lepetit T., Gu Q., Bahari B., Fainman Y., Kante B. (2017). Lasing action from photonic bound states in continuum. Nature.

[8] Zhang X., Liu Y., Han J., Kivshar Y., Song Q. (2022). Chiral emission from resonant metasurfaces. Science.

[9] Wang J., Kühne J., Karamanos T., Rockstuhl C., Maier S. A., Tittl A. (2021). All‐dielectric crescent metasurface sensor driven by bound states in the continuum. *Adv. Funct. Mater.*.

[10] Karawdeniya B. I., Damry A. M., Murugappan K. (2022). Surface functionalization and texturing of optical metasurfaces for sensing applications. *Chem. Rev.*.

[11] Yesilkoy F., Arvelo E. R., Jahani Y. (2019). Ultrasensitive hyperspectral imaging and biodetection enabled by dielectric metasurfaces. Nat. Photonics.

[12] Bernhardt N., Koshelev K., White S. J. U. (2020). Quasi-BIC resonant enhancement of second-harmonic generation in WS_2_ monolayers. Nano Lett..

[13] Xu L., Zangeneh Kamali K., Huang L. (2019). Dynamic nonlinear image tuning through magnetic dipole quasi-BIC ultrathin resonators. Adv. Sci..

[14] Liu Z., Xu Y., Lin Y. (2019). High-Q quasibound states in the continuum for nonlinear metasurfaces. Phys. Rev. Lett..

[15] Zhang X., He L., Gan X. (2022). Quasi-bound states in the continuum enhanced second-harmonic generation in thin-film lithium niobate. Laser Photonics Rev..

[16] Li S., Zhou C., Liu T., Xiao S. (2019). Symmetry-protected bound states in the continuum supported by all-dielectric metasurfaces. Phys. Rev. A.

[17] Jin J., Yin X., Ni L., Soljačić M., Zhen B., Peng C. (2019). Topologically enabled ultrahigh-Q guided resonances robust to out-of-plane scattering. Nature.

[18] Yin X., Jin J., Soljačić M., Peng C., Zhen B. (2020). Observation of topologically enabled unidirectional guided resonances. Nature.

[19] Kang M., Zhang Z., Wu T. (2022). Coherent full polarization control based on bound states in the continuum. Nat. Commun..

[20] Fan K., Shadrivov I. V., Padilla W. J. (2019). Dynamic bound states in the continuum. Optica.

[21] Vaity P., Gupta H., Kala A. (2021). Polarization-independent quasibound states in the continuum. *Adv. Photonics Res.*.

[22] Murai S., Abujetas D. R., Liu L. (2022). Engineering bound states in the continuum at telecom wavelengths with non-bravais lattices. *Laser Photonics Rev.*.

[23] Tian J., Li Q., Belov P. A., Sinha R. K., Qian W., Qiu M. (2020). High-*Q* all-dielectric metasurface: super and suppressed optical absorption. ACS Photonics.

[24] Zhou C., Huang L., Jin R. (2023). Bound states in the continuum in asymmetric dielectric metasurfaces. Laser Photonics Rev..

[25] Kang M., Zhang S., Xiao M., Xu H. (2021). Merging bound states in the continuum at off-high symmetry points. Phys. Rev. Lett..

[26] Zhou Q., Fu Y., Liu J. (2022). Plasmonic bound states in the continuum in compact nanostructures. *Adv. Opt. Mater.*.

[27] Zhou Y., Guo Z., Zhao X. (2022). Dual-quasi bound states in the continuum enabled plasmonic metasurfaces. Adv. Opt. Mater..

[28] Wang X., Wang J., Zhao X., Shi L., Zi J. (2022). Realizing tunable evolution of bound states in the continuum and circularly polarized points by symmetry breaking. ACS Photonics.

[29] Wang B., Liu W., Zhao M. (2020). Generating optical vortex beams by momentum-space polarization vortices centred at bound states in the continuum. Nat. Photonics.

[30] Liu W., Wang B., Zhang Y. (2019). Circularly polarized states spawning from bound states in the continuum. Phys. Rev. Lett..

[31] Hsu C. W., Zhen B., Stone A. D., Joannopoulos J. D., Soljačić M. (2016). Bound states in the continuum. Nat. Rev. Mater..

[32] Sadreev A. F. (2021). Interference traps waves in an open system: bound states in the continuum. Rep. Prog. Phys..

[33] Bulgakov E. N., Sadreev A. F. (2019). High-Q resonant modes in a finite array of dielectric particles. Phys. Rev. A.

[34] Huang L., Xu L., Rahmani M., Neshev D., Miroshnichenko A. E. (2021). Pushing the limit of high-Q mode of a single dielectric nanocavity. *Adv. Photonics*.

[35] Al-Ani I. A. M., As’Ham K., Huang L., Miroshnichenko A. E., Hattori H. T. (2021). Enhanced strong coupling of TMDC monolayers by bound state in the continuum. Laser Photonics Rev..

[36] Overvig A., Yu N., Alu A. (2021). Chiral quasi-bound states in the continuum. Phys. Rev. Lett..

[37] Yu J., Ma B., Qin R., Ghosh P., Qiu M., Li Q. (2022). High-Q absorption in all-dielectric photonics assisted by metamirrors. ACS Photonics.

[38] Shi T., Deng Z.-L., Tu Q.-A., Cao Y., Li X. (2021). Displacement-mediated bound states in the continuum in all-dielectric superlattice metasurfaces. *PhotoniX*.

[39] He Y., Guo G., Feng T., Xu Y., Miroshnichenko A. E. (2018). Toroidal dipole bound states in the continuum. *Phys. Rev. B*.

[40] Yu J., Ma B., Ouyang A. (2021). Dielectric super-absorbing metasurfaces via PT symmetry breaking. Optica.

[41] Bulgakov E. N., Sadreev A. F. (2014). Bloch bound states in the radiation continuum in a periodic array of dielectric rods. Phys. Rev. A.

[42] Pankin P. S., Wu B.-R., Yang J.-H., Chen K.-P., Timofeev I. V., Sadreev A. F. (2020). One-dimensional photonic bound states in the continuum. Commun. Phys..

[43] Koshelev K., Lepeshov S., Liu M., Bogdanov A., Kivshar Y. (2018). Asymmetric metasurfaces with high-Q resonances governed by bound states in the continuum. Phys. Rev. Lett..

[44] Han S., Pitchappa P., Wang W., Srivastava Y. K., Rybin M. V., Singh R. (2021). Extended bound states in the continuum with symmetry-broken terahertz dielectric metasurfaces. Adv. Opt. Mater..

[45] Li Z., Zhou L., Liu Z. (2022). Modifying the quality factors of the bound states in the continuum in a dielectric metasurface by mode coupling. *ACS Photonics*.

[46] Campione S., Liu S., Basilio L. I. (2016). Broken symmetry dielectric resonators for high quality factor Fano metasurfaces. ACS Photonics.

[47] Cui C., Zhou C., Yuan S. (2018). Multiple Fano resonances in symmetry-breaking silicon metasurface for manipulating light emission. ACS Photonics.

[48] Hu L., Wang B., Guo Y. (2022). Quasi-BIC enhanced broadband terahertz generation in all-dielectric metasurface. Adv. Opt. Mater..

[49] Xu L., Rahmani M., Chiang Y. K. (2020). Enhanced light–matter interactions in dielectric nanostructures via machine-learning approach. *Adv. Photonics*.

[50] Sinev I. S., Koshelev K., Liu Z. (2021). Observation of ultrafast self-action effects in quasi-BIC resonant metasurfaces. Nano Lett..

[51] Zhou C., Qu X., Xiao S., Fan M. (2020). Imaging through a fano-resonant dielectric metasurface governed by quasi--bound states in the continuum. Phys. Rev. Appl..

[52] Overvig A. C., Shrestha S., Yu N. (2018). Dimerized high contrast gratings. Nanophotonics.

[53] Johnson S. G., Joannopoulos J. D. (2001). Block-iterative frequency-domain methods for Maxwell’s equations in a planewave basis. Opt. Express.

[54] Miroshnichenko A. E., Flach S., Kivshar Y. S. (2010). Fano resonances in nanoscale structures. Rev. Mod. Phys..

[55] Lim W. X., Manjappa M., Pitchappa P., Singh R. (2018). Shaping high-*Q* planar Fano resonant metamaterials toward futuristic technologies. Adv. Opt. Mater..

[56] Kaelberer T., Fedotov V. A., Papasimakis N., Tsai D. P., Zheludev N. I. (2010). Toroidal dipolar response in a metamaterial. Science.

[57] Gupta M., Savinov V., Xu N. (2016). Sharp toroidal resonances in planar terahertz metasurfaces. Adv. Mater..

[58] Ahmadivand A., Gerislioglu B., Manickam P. (2017). Rapid detection of infectious envelope proteins by magnetoplasmonic toroidal metasensors. ACS Sens..

[59] Gerislioglu B., Ahmadivand A., Pala N. (2018). Tunable plasmonic toroidal terahertz metamodulator. Phys. Rev. B.

[60] Qin P., Yang Y., Musa M. Y. (2018). Toroidal localized spoof plasmons on compact metadisks. Adv. Sci..

[61] Jeong J., Goldflam M. D., Campione S. (2020). High quality factor toroidal resonances in dielectric metasurfaces. ACS Photonics.

[62] Zhou C., Li S., Wang Y., Zhan M. (2019). Multiple toroidal dipole Fano resonances of asymmetric dielectric nanohole arrays. Phys. Rev. B.

[63] Song D., Wang H., Deng M., Wang Y. (2021). Toroidal dipole Fano resonances supported by lattice-perturbed dielectric nanohole arrays in the near-infrared region. Appl. Opt..

[64] Yuan L., Jeong J., Chi Kwock K. W. (2021). Manipulation of exciton dynamics in single-layer WSe_2_ using a toroidal dielectric metasurface. Nano Lett..

[65] Li S., Li S., Wang Y. (2022). Near-infrared toroidal dipole response supported by silicon metasurfaces. Appl. Opt..

[66] Wu P. C., Liao C. Y., Savinov V. (2018). Optical anapole metamaterial. ACS Nano.

